# Tidal Variations of Fish Larvae Measured Using a 15-Day Continuous Ichthyoplankton Survey in Subei Shoal: Management Implications for the Red-Crowned Crane (*Grus japonensis*) Population in Yancheng Nature Reserve

**DOI:** 10.3390/ani13193088

**Published:** 2023-10-03

**Authors:** Min Xu, Zunlei Liu, Xiaojing Song, Fei Wang, Yihe Wang, Linlin Yang, Takayoshi Otaki, Jiabin Shen, Teruhisa Komatsu, Jiahua Cheng

**Affiliations:** 1 Key Laboratory of East China Sea and Oceanic Fishery Resources Exploitation, Ministry of Agriculture, No 300, Jungong Road, Shanghai 200090, China; xuminwzy@aliyun.com (M.X.); liuzl@ecsf.ac.cn (Z.L.); songxiaojing@ecsf.ac.cn (X.S.); wangfei@ecsf.ac.cn (F.W.); 2 East China Sea Fisheries Research Institute, Chinese Academy of Fishery Sciences, Shanghai 200090, China; shenjb@ecsf.ac.cn; 3 State Key Laboratory of Estuarine and Coastal Research, East China Normal University, Shanghai 200241, China; yhwang@sklec.ecnu.edu.cn; 4 School of Marine Sciences, East China Normal University, Shanghai 200241, China; 5 Japan Fisheries Information Service Center, Tokyo 104-0055, Japan; otaki@jafic.or.jp; 6 Japan Fisheries Resource Conservation Association, Tokyo 104-0044, Japan; cymodocea@gmail.com

**Keywords:** birds nature reserve, *Grus japonensis*, ichthyoplankton survey, Subei Shoal, tide, *Acanthogobius ommaturus*, *Lateolabrax japonicus*, *Liza haematocheila*

## Abstract

**Simple Summary:**

The red-crowned crane *Grus japonensis* in the National Yancheng Rare Birds Nature Reserve needs large quantities of high-protein food sources, including juvenile fishes, to fuel their migratory journeys. Some fish species use the tide to complete inshore migration, and they gradually migrate from the open sea to the surf area to find a more suitable habitat. In this paper, we used a fixed station to assess how the number of characteristics and developmental stages of fish larvae vary with the tide and how these values are related to environmental variables. We found that the number of species and larval individuals were highest and lowest, respectively, at the highest and lowest tidal height, and they obviously increased and decreased with the rising and ebb tide, respectively. We detected a tendency for *Cynoglossus joyneri*, *Larimichthys polyactis*, and *Liza haematocheila* to be present at higher density during the rising tide compared with the ebb tide, indicating that they were moving to the near shore from the open sea. Our findings indicate that the variation in numbers of the larvae and juveniles depends on species and developmental stage. These results provide a better understanding of the habitat of prey species of the wild bird population.

**Abstract:**

The National Yancheng Rare Birds Nature Reserve is a vitally important staging habitat for the wild population of red-crowned cranes (*Grus japonensis*) in China. The population relies on local high-protein food sources, such as fish juveniles, to fuel their migratory journeys. However, little is known about the ecology of the fish larvae and juveniles that migrate to the inshore area via tidal rhythm in Subei Shoal, which is adjacent to the reserve. Therefore, we used a fixed study station (32°55′1.2″ N, 121°19′58.8″ E) to conduct a continuous 15-day ichthyoplankton survey at 2 h intervals beginning at 05:00 on 25 April and ending at 03:00 on 10 May 2019. We identified the tidal variations in the number of fish larvae and juveniles and the number at various developmental stages and assessed how they were related to environmental variables such as sea surface temperature, salinity, turbidity, and tidal height in the Dafeng Sea area of Subei Shoal. We found that the number of species and larval individuals were highest and lowest, respectively, at the highest and lowest tidal height, and they obviously increased and decreased with the rising and ebb tide, respectively. Our findings indicate that the variation in numbers of the larvae and juveniles depends on species and developmental stage. The species *Acanthogobius ommaturus*, *Pholis fangi*, *Cynoglossus joyneri*, *Liza haematocheila,* and *Lateolabrax japonicus* and the total number of larvae were most influenced by tidal height. These results provide a better understanding of the habitat of prey species of the red-crowned crane wild population as well as scientific data that can be applied to manage the wild population in the reserve sustainably.

## 1. Introduction

The National Yancheng Rare Birds Nature Reserve (NYBNR) was established in 1983, and it is the largest coastal nature reserve in China for the conservation of the red-crowned crane (*Grus japonensis*) [1]. Globally, this is an endangered migratory water bird species that inhabits wetlands. The reserve extends for 582 km along the coastline of the Yellow Sea [2]. It became an international biosphere reserve in UNESCO’s Man and the Biosphere Programme in 1992 and was included in the Ramsar Convention List of Wetlands of International Importance in 2002 [3]. The reserve adjacent to the Subei Shoal area is a critical habitat for 405 bird species, including the red-crowned crane, and approximating 3,000,000 migratory birds annually [4]. In China, the red-crowned crane has been classified as a national first-grade protected bird, and migration staging sites such as the reserve are vital for its wild population [5]. Red-crowned cranes require large quantities of high-protein food sources, such as juvenile fish, to fuel their migratory journeys [6,7]. Li et al. (2014) detected fish remains in their fecal samples [8].

Some fish species used the tide to complete inshore migration. Fishes gradually migrate from the open sea to the surf area or the inner bay to find a more suitable habitat [9]. The tidal creek area of Subei Shoal is connected to the open sea, and fish in the open sea can move along the tidal creek to the near shore. Yan et al. (2016) classified the fish community in the Dafeng coastal waters of Subei Shoal into three communities: the western coasts, the central sand ridge, and the eastern tidal creek. In spring, the main fish species of the western coasts were *Pholos fangi*, *Coilia mystus*, and *Miichthys miiuy*; those of the central sand ridge were *Thryssa kammalensis*, *Cynoglossus lighti*, and *P. fangi*; and those of the eastern tidal creek were *Eupleurogrammus muticus* and *P. fangi*. They calculated the number percentage of fish juveniles in the total number and biomass density in a square kilometer for *P. fangi* and *C. lighti* to be 89.55% with 2.8 kg km^−2^ and 66.67% with 4.76 kg km^−2^ in the Dafeng coastal waters of Subei Shoal in May 2011 [10]. Xiong et al. (2007) investigated fish egg distribution in Subei Shoal in late May of 2004 and 2005, and the dominant species were *Sillago sihama*, *Engraulis japonicus*, *Konosirus punctatus*, *Sardinops melanostictus*, and *Ilisha elongata* [11]. Liu and Guo et al. (2009) described Dafeng coastal waters of Subei Shoal in late May of 2006 and 2007 as the nursery ground for *K. punctatus*, *Liza haematochelia*, *Larimichthys crocea*, and *Nibea mitsukurii* [12]. However, how these fish larvae and juveniles migrate to the coastal area, including the NYBNR, via the tides and the relationships between the fish and the red-crowned crane are not well understood.

The distribution of fish larvae in the northern part of Subei Shoal is determined by the strength of water masses such as Jiangsu surface runoff, Changjiang diluted water, coastal currents in the East China Sea and the Yellow Sea, and the Yellow Sea cold water mass [13]. Zeng et al. (2020) found that the monthly average density and catches of fish larvae in the southern branch of the Yangtze River Estuary were affected by different degrees of flood and ebb tide from May 2017 to April 2018. During the monthly spring tide, the body length and developmental stages of *Hemiculter bleekeri*, *L. haematocheila*, *Culter alburnus*, *Rhinogobius giurinus*, and *Repomucenus olidus* changed with the tides [14]. Chang et al. (2015) found that the species number of larvae and the individual number of *Pseudogobius javanicus* larvae differed significantly with the tidal level (high tide or low tide), and the individual number of *Omobranchus elegans* differed significantly with the tidal type (diurnal tide and semidiurnal tide) [15]. Zhang et al. (2020) reported that the species number of larvae increased with the increase in tidal level and that the tides in the Yangtze River Estuary have a greater impact on larval abundance and a lesser impact on the number of species, as the abundance decreased at high tide and increased at low tide [16]. Although Kang et al. (2013) and Tian et al. (2011) studied the distribution of plankton abundance in the sand ridges [17] and tidal creeks of the radial sandbank area of Subei Shoal [18], to date, there is scant knowledge about how fish larvae numbers vary with the tides in Subei Shoal.

The Subei Shoal area is historically regarded as the nursery and spawning ground for fish species, which was important for the existence of the Lvsi fishing ground [10,19]. Yan et al. (2016) identified the group of adult spawning fishes in the mouth of the tidal creek in spring [20], and Xu and Wang et al. (2014) speculated that groups of spawning fishes from the open sea entered into the shoal area to spawn in May [19]. In this study, we used a fixed study station to assess how the number of characteristics and developmental stages of fish larvae vary with the tidal rhythm (day against night, rising tide against ebb tide) and how these values are related to environmental variables such as sea surface temperature, salinity, turbidity, and tidal height beginning. We are the first to report how the numbers of fish larvae vary with environmental conditions in a long-term 15-day tidal cycle (25 April to 10 May 2019) in this area. Our results provide a better understanding of the origin of food, especially fish juveniles and larvae, required by the red-crowned crane and can be applied to accurately estimate the fish food capacity of coastal areas in the NYBNR.

## 2. Materials and Methods

The study was approved by the ethics committee of the East China Sea Fisheries Research Institute, Chinese Academy of Fishery Sciences. The surveys comply with the current laws of China. All of the ichthyoplankton samples in this study were obtained from legal fisheries actions. All procedures were performed following the guidelines of the American Fisheries Society for the use of fishes in research. It did not involve any endangered or protected species listed in the China Red Data Book of Endangered Animals.

**Figure 1:**
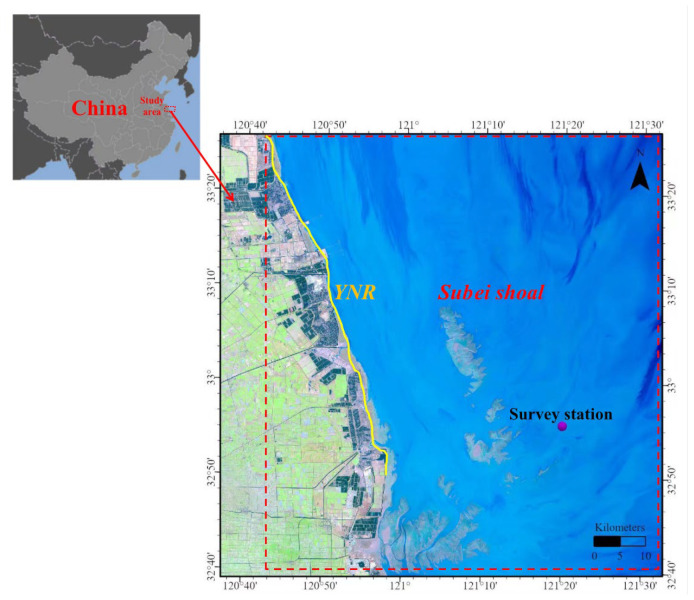
Map showing the survey station (32°55′1.2″ N, 121°19′58.8″ E; denoted by a purple solid dot) in the Subei Shoal (denoted by the red dotted line frame) adjacent to the Yancheng Nature Reserve (YNR) (denoted by yellow solid line) in China.

Our study station (32°55′1.2″ N, 121°19′58.8″ E) was located in Subei Shoal in a strong tidal area that is influenced by the tidal creek system (Figure 1). Subei Shoal is located in the southwest of the South Yellow Sea and is the largest muddy coastal tidal flat wetland in Asia [21]. The seabed topography is composed of tidal creeks and sand ridges that extend 200 km from north to south and 90 km from east to west [22]. The width of the sand ridge gradually widens, and the tidal creek gradually becomes narrow and shallow from the open sea to the coast, extending outward in a radiating shape [22].

The Subei Shoal area is adjacent to the Yancheng Dafeng red-crowned crane nature reserve, and more than 60% of the world’s wild red-crowned crane population spends winters there (Figure 1) [21]. The southwest and southeast parts of the study area are strongly influenced by the Yangtze River and the Kuroshio Warm Current [19]. The mean tidal range of the study area is ~4 m, and the average depths of the tidal creek and sand ridge are 13.6 m and 4.72 m, respectively [23].

**Figure 2:**
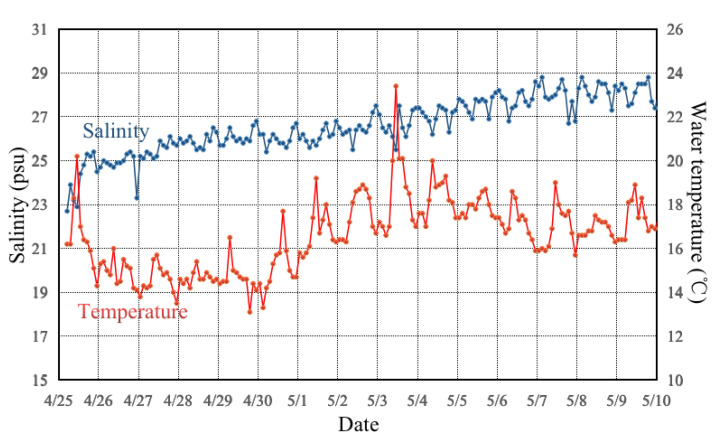
Temporal variations of water temperature (°C) and salinity (psu) denoted by the scarlet and blue lines, respectively, during the survey conducted from 25 April to 10 May 2019.

In this study, we used a fishing boat (#SUDONGTAI 18068, 19.90 m length, gross tonnage of 46 t, and main engine gross power of 73.0 kw) to perform a continuous 15-day ichthyoplankton survey at 2 h intervals. We conducted 180 hauls from 05:00 on 25 April to 03:00 on 10 May 2019 at one fixed station in the Dafeng Sea area of the Subei Shoal (Figure 1). The salinity increased from 22.7 to 28.3 psu over the course of the study (Figure 2).

To collect ichthyoplankton samples, the survey boat was stopped, and a cone-shaped ichthyoplankton net (130 cm diameter, 600 cm length, and 0.505 mm mesh size) was cast into the sea surface. It was equipped with a calibrated flowmeter mounted in the center of the net mouth to measure the flow rate. For each haul, near-surface sampling was carried out with the top of the net ring just below the air–water interface for 10 min. After the haul, ichthyoplankton samples were immediately washed into a stainless end collection cup with flowing seawater. The samples were preserved in situ in 5% buffered formaldehyde prepared in seawater for further analysis. Larvae from the samples were identified using morphological classification and enumerated in the laboratory using a stereomicroscope (ZEISS, Stemi 2000, Oberkochen, Germany). The developmental stages were classified into the yolk-sac, preflexion, flexion, postflexion, and juvenile stages. The larval density in the ichthyoplankton samples was quantitatively converted to the number per 100 m^3^ filtered seawater volume (inds 100 m^−3^). We used a water quality measuring device (Hydrolab HL7, HACH, Shanghai, China) during each haul to record sea surface temperature (SST; °C), sea surface salinity (SSS; psu), and sea surface turbidity (NTU) data, and we checked and recorded the tidal height (cm) corresponding to the time of each haul from the China tide table book for 2019. We performed Tukey tests on the number of species and larval number according to moon positions relative to Earth, including the third quarter, apogee, smallest red latitude, the first day of the lunar month, and the position northernmost of the equator. We performed analysis of variance tests on larval density and number according to the rising tide and ebb tide.

The habitat suitability index (*HSI*) was proposed by the U.S. Fish and Wildlife Service in the 1980s to assess the habitat quality of wild animals and predict their environmental distribution, and it has been especially applicable in studies of fishes. The Service created 157 models for wild birds and fishes. In this study, we calculated the *HSI* to investigate the total species number, total individual number, and the density (inds 100 m^−3^) in each haul and evaluated their relation to the selected hydrographic parameters. We also investigated the relationships between the total individual number and larval density of *L. japonicus*, *A. ommaturus*, *C. joyneri*, *L. haematocheila*, and *P. fangi* in each haul and the four hydrographic parameters during the survey period. *HSI* was obtained by comprehensive calculations of many number-based suitability index (SI) values, which were estimated as follows:

*SI* = (*Y* − *Y*_min_)/(*Y*_max_ − *Y*_min_) (1)

where *Y* is the number after smoothed regression, and *Y*_max_ and *Y*_min_ are the maximum and minimum predicted values, respectively. Each *SI* was estimated as a value between 0.0 and 1.0. *SI* values between 0.7 and 1.0 correspond to environmental factors that are in the most suitable environment range [24].

We calculated *HSI* values using the equation given below:

(2) $$HSI=\frac{1}{\sum_{i=1}^{n}w_{i}}\cdot \sum_{i=1}^{n}{SI}_{i}w_{i}$$

where *SI_i_* is the *SI* value of the environmental variable, *i* and *w_i_* are the weight of the environmental variable, and *i* and *n* are the number of environmental factors [24].

## 3. Results and Discussion

Analysis of variance revealed no significant differences in larval number and density among the groups of the rising tide and ebb tide. However, we detected significant differences between the northernmost of the equator group and the third quarter, apogee, smallest red latitude, and first day of the lunar month groups and between the first day of the lunar month and the third quarter and apogee groups via Tukey tests.

**Figure 3:**
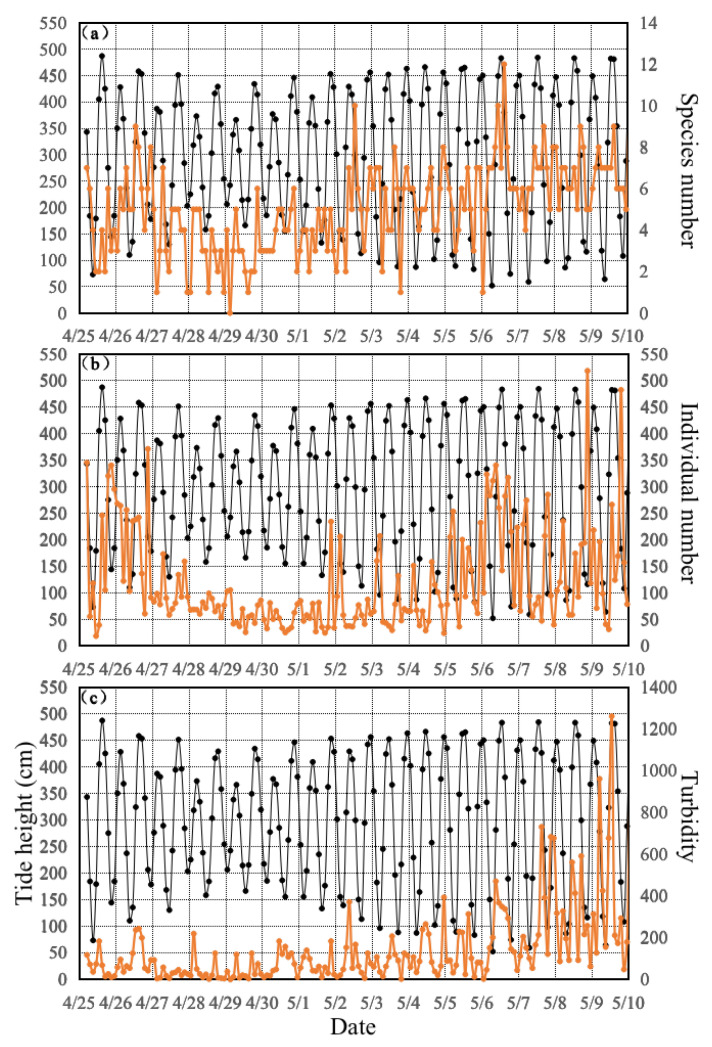
Temporal variations of (**a**) species number (num), (**b**) individual number (inds), and (**c**) turbidity (NTU) relative to tidal height (cm) denoted by the scarlet (**a**–**c**) and black lines (tidal height) during the survey conducted from 25 April to 10 May 2019.

The fish species number and number of larval individuals were the most and least abundant at the highest and lowest tidal heights, respectively. They obviously increased and decreased with the rising and ebb tide, respectively, in the cases of 5:00–9:00 on 25 April, 11:00–17:00 on 25–26 April, 3:00–7:00 on 29 April, and 15:00–19:00 on 6 May (Figure 3). Hare et al. (2005) suggested the presence of selective tidal stream transport whereby larvae are more abundant during the rising tide and less abundant during the ebb tide. Such tidal behaviors are expressed as larvae moving higher in the water column on flood tides and lower in the water column on the ebb tide, resulting in upcoast transport [25]. Joyeux (1999) concluded that Atlantic menhaden (*Brevoortia tyrannus*), Atlantic croaker (*Micropogonias undulatus*), summer flounder (*Paralichthys dentatus*), and pinfish (*Lagodon rhomboides*) larvae enter the Beaufort Inlet in North Carolina as a result of selective tidal stream transport [26]. Forward et al. (1998) and Burke et al. (1998) demonstrated that late-stage fish larvae may have an entrained tidal rhythm of behavior [27,28]. Rowe and Epifanio (1994) and Churchill et al. (1999) found that the upestuary transport of weakfish (*Cynoscion regalis*) larvae was associated with the flow speed [29,30]. Tzeng and Wang (1992) reported increased food and range of larval activity during the flood tide and the opposite trend during ebb tide [31]. Hou et al. (2018) found that the species number and abundance in the rising tide were greater than those of the ebb tide in the Yangtze River mouth during each survey month [32]. Tidal patterns may also influence the larval distribution.

In contrast, we found the highest numbers of species and larval individuals during the middle of the tides, such as the cases of 17:00–21:00 on 25 April, 5:00–19:00 on 26 April, 11:00–17:00 on 1 May, 11:00–17:00 on 2 May, and 15:00–21:00 on 8 May (Figure 3). We assumed that the highest zooplankton abundance occurred during these periods to account for this phenomenon. Yan and Xu et al. (2016) reported a large number of larvae and high zooplankton abundance in the tidal creek of Subei Shoal [20]. The plumes controlled by the tide form distinct frontal boundaries that can alter the spatial distributions of larval fishes and their planktonic prey and predators [33]. The freshwater plume provided substantially higher concentrations of prey and promoted spatial overlap between the prey and fish larvae. This finding suggested a close relationship between the larvae and zooplankton abundance. More detailed surveys of the fine-scale spatial distributions of larval fishes, their prey, and their predators are needed to understand the mechanisms of larval success and their relation to physical processes.

**Figure 4:**
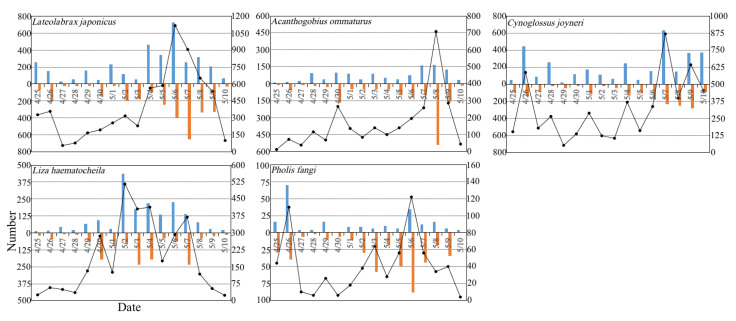
Variations in the number of fish during the rising, ebb, and one whole tide denoted by the blue bar, scarlet bar, and black line, respectively, for *Lateolabrax japonicus*, *Acanthogobius ommaturus*, *Cynoglossus joyneri*, *Liza haematocheila*, and *Pholis fangi* during the survey conducted from 25 April to 10 May 2019.

For the fish species *Lateolabrax japonicus*, *Acanthogobius ommaturus*, *Cynoglossus joyneri*, *L. haematocheila*, and *Pholis fangi*, the largest differences in the number of individuals between the rising and ebb tides were −394 inds on 7 May, −372 inds on 8 May, 396 inds on 7 May, 352 inds on 2 May, and −54 inds on 6 May, respectively (Figure 4). A negative value means that the number during the ebb tide was greater than that during the rising tide. The number percentages of *L. japonicus*, *C. joyneri*, *L. haematocheila*, *P. fangi*, and *Neosalanx anderssoni* were 76.63% vs. 23.37%, 25.14% vs. 74.86%, 64.33% vs. 35.67%, 64.04% vs. 35.95%, and 84.61% vs. 15.38%, respectively, during the day and night (Table 1). One explanation for the observed diel variation in abundance is net avoidance, which is based on the assumption that larger larvae are more able than smaller larvae to avoid capture in the net during the day because they are stronger swimmers and have a better visual sense.

The number of *L. japonicus* individuals was 328–358 on 25–26 April, and then it increased to 1116 inds on 6 May, which was the first day of a lunar month, and then decreased to 100 inds on 10 May (Figure 4). The number percentage of *L. japonicus* juveniles was higher in the rising tide during the day (43.76% vs. 32.87%) and the ebb tide during the night (10.15% vs. 13.22%) (Table 1). The feeding habits of *L. japonicus* might influence the density distribution of the larvae during the day and night. Larvae have weak night vision and are unable to feed in the darkness at night, so they prefer to feed in the day [34,35,36]. Trnski (2001) argued that the diel phase had a stronger influence on the abundance of the most common taxa and community structure [37].

The two peaks of *A. ommaturus* larvae number were 266 inds on 30 April and 708 inds on 8 May, which shows a 7-day interval (Figure 4). The number percentages of *A. ommaturus* (28.91% vs. 24.52% during the day and 27.86% vs. 18.70% at night) and *P. fangi* (40.48% vs. 23.56% during the day and 26.28% vs. 9.67% at night) during the ebb tide were higher than those during the rising tide in the day and night (Table 1). Liu et al. (2016) also reported greater densities of *A. ommaturus* larvae during the rising tide in the southeast area of the Yangtze Estuary and lower densities during the ebb tide in the southern area [38]. In addition, the number peaks of *P. fangi* occurred on 26 April with 110 inds and on 6 May with 122 inds (Figure 4). The order of the number percentage of *P. fangi* larvae was as follows: ebb tide during the day (40.48%) > ebb tide at night (26.28%) > rising tide during the day (23.56%) > rising tide at night (9.67%) (Table 1). This pattern would reduce predation risk or increase prey densities if larvae are near the surface at night [39]. Neira and Potter (1992) examined diel and tidal variation in larval fish abundance in barrier-estuary channels and argued that larvae of many taxa were more abundant at night [40]. Yan and Xu et al. (2016) identified the tidal creek of Subei Shoal as mainly the feeding ground of *P. fangi* young fish, most of which were found in the tidal creek in the middle sea area of Subei Shoal. *P. fangi* was the main dominant species in Subei Shoal in the spring, accounting for 38.04% of the total fish biomass in this area [20].

The coastal species *C. joyneri* is the main target species for coastal deep-water stow net fisheries, which has a stable fishery production each year in the Jiangsu Lvsi area. *C. joyneri* prefer to inhabit 0–20 m depth and mainly feed on benthic organisms such as polychaetes and cumaceans in muddy areas [41]. In our study, more larval individuals of *C. joyneri* were found on 26 (586 inds) and 28 April (264 inds) and 1 (288 inds), 4 (368 inds), 7 (868 inds), and 9 (642 inds) May (Figure 4). The postflexion larvae of *C. joyneri* were dominant in the rising tide at night (49.82%) (Table 1).

The number peaks of *L. haematocheila* larvae occurred on 30 April (286 inds) and 2 (516 inds), 4 (414 inds), and 7 (370 inds) May (Figure 4). The individual percentage of the larvae was higher during the rising tide than during the ebb tide (55.96% vs. 44.04%). Larvae of marine-spawned fishes have to exploit flood-tide flux to enter estuaries [42]. Zeng et al. (2020) found that the individual number and mean density of preflexion stage larvae of this species were higher during the rising tide than the ebb tide [14]. *L. haematocheila* is a eurythermal and euryhaline coastal species that generally inhabits estuaries and bays. They usually spawn 2–8 km away from the shore [13]. Jiang et al. (2007) used a plankton net with 80 cm diameter and 0.505 mm mesh size to identify the horizontal distribution of *L. haematocheila* fish larvae from 22 to 28 May 2005. They found that 4–5 mm was the dominant body length (range, 2–23 mm), and flexion larvae constituted 58.8% of the total number. Jiang et al. (2007) suggested that there was a spawning ground for this species near the north of Subei Shoal and that the eggs would drift from north to south with the current [13]. However, similar to Liu et al. (2009), we found no eggs in surveys conducted in late April to early May of 2006, 2007, and 2019, which indicates the important role of tidal current transport [12]. Zhong et al. (2006) identified the surf zone along the Yangtze River coast as a good nursery ground for *L. haematocheila* larvae. Its developmental stages were found relatively early in the open sea, whereas later stages were found in the coastal area, indicating the tendency to migrate to coastal areas with the tidal current movement [43]. Larval buoyancy may generate tidal patterns in larval distribution, with higher number densities in the shallower water column and lower densities in the deeper water [44].

We also found that the order of number percentages for the yolk-sac and preflexion stages of *L. haematocheila* larvae varied as follows: rising tide during the day (34.82%) > ebb tide during the day (29.51%) > rising tide at night (21.14%) > ebb tide at night (14.53%) (Table 1). The number percentages of *L. haematocheila* and *Mugil cephalus* juveniles at night were greater than those during the day (29.25% vs. 7.46% and 71.88% vs. 28.13%) (Table 1), indicating the possibility of night feeding for individuals at later developmental stages with better swimming ability [14]. Large diel differences in the abundance of larvae in Lake Macquarie have been reported, with many taxa being more abundant at night than during the day [45].

**Figure 5:**
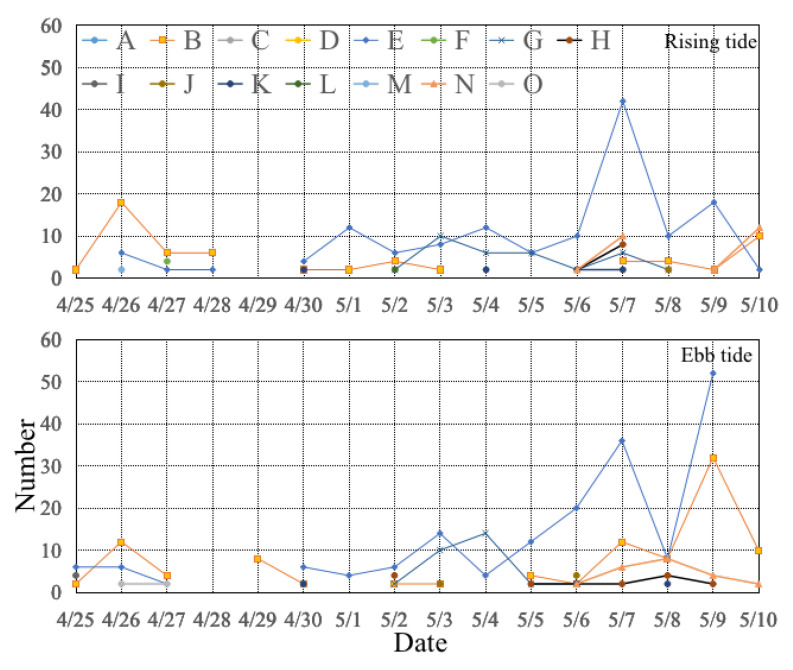
Variations in the number of fish during the rising and ebb tide for (A) *Acanthopagrus schlegelii*, (B) *Amblychaeturichthys hexanema*, (C) *Ammodytes personatus*, (D) *Anguilla japonica*, (E) *Callionymus* sp., (F) *Clupea pallasii*, (G) *Mugil cephalus*, (H) *Neosalanx anderssoni*, (I) *Omobranchus elegans*, (J) *Ophichthyidae* sp., (K) *Platycephalus indicus*, (L) *Scomberomorus niphonius*, (M) *Stolephorus* sp., (N) *Takifugu* sp., and (O) *Trypauchen vagina* during the survey conducted from 25 April to 10 May 2019.

The species present with the lowest abundance were *Acanthopagrus schlegelii*, *Ammodytes personatus*, *Anguilla japonica*, *Clupea pallasii*, *N. anderssoni*, *O. elegans*, *Ophichthyidae* spp., *Platycephalus indicus*, *Scomberomorus niphonius*, *Stolephorus* sp., and *Trypauchen vagina* (Figure 5). The larval number of *Amblychaeturichthys hexanema* and *Callionymus* sp. during the ebb tide was greater than that during the rising tide (100 > 62 inds and 176 > 140 inds, respectively) (Figure 5). The abundance of juvenile *Callionymus* sp. was highest at ebb tide during the day (15.19%). The larvae of *N. anderssoni* were most numerous at rising tide during the day (38.46%). The number percentages of *P. indicus* at later developmental stages of postflexion and juveniles were close to each other (42.86% vs. 57.14%). The order of number percentages of *M. cephalus* juveniles was as follows: rising tide at night (46.88%) > ebb tide at night (25%) > ebb tide during the day (21.88%) > rising tide during the day (6.25%) (Table 1). The southern distribution limit of the small fish species *A. personatus* is the Yellow Sea in the Northwest Pacific. They burrow into the sand from summer to autumn and migrate a very short distance away from the spawning location to release eggs simultaneously at one time [46]. The Japanese eel *A. japonica*, with a mean body length of 56.1 mm and a full-length range of 42.0–68.0 mm, is a main fishing target from February to April in Jiangsu coastal areas of China [47].

In addition, we found that the developmental stages of *A. ommaturus*, *A. hexanema*, *Callionymus* sp., *C. joyneri*, *L. haematocheila*, and *P. indicus* larvae were dominated, respectively, by flexion (26.74%) to postflexion (68.23%) (preflexion to juvenile); postflexion (43.21%) to juvenile (55.56%) (postflexion to young fish); postflexion (68.98%) to juvenile (25.95%) (preflexion to juvenile); postflexion (97.37%) (preflexion to juvenile); preflexion (52.52%) and juvenile (36.71%) (yolk-sac to juvenile); and postflexion (42.87%) to juvenile (57.13%) (Table 1). Liu et al. (2009) reported the size and density of the postflexion stage of *A. ommaturus* to be 12.0 mm body length and 0.1 inds m^−3^, those of the preflexion stage of *C. joyneri* to be 5.9 mm body length and 0.1 inds m^−3^, those of the yolk-sac to the postflexion stage of *L. haematocheila* to be 1.9–6.5 mm body length and 39.1 inds m^−3^, and those of the preflexion to the postflexion stage of *P. indicus* to be 4.9–5.4 mm body length and 0.3 inds m^−3^ in Subei Shoal from 2006 to 2007 [12].

In our study, we detected a tendency for *C. joyneri*, *Larimichthys polyactis*, and *L. haematocheila* to be present at higher density during the rising tide compared with the ebb tide (Table 1), indicating that they were moving to the near shore from the open sea. Liu et al. (2016) found more fish larvae in the rising tide compared with the ebb tide in May in the Yangtze River mouth [38]. Xu et al. (2023) reported two distinct early life stage distribution patterns of *L. polyactis*: one occurring during March to April in the deep waters seaward of the Changjiang River bottom plume front and the other occurring in May to June throughout the entire water column in coastal waters. They also suggested a potential fish larval migration route from the southern deep shelf waters to the northern coastal waters within the motor-trawl prohibition line [48]. Lin et al. (2018) found that postflexion larvae and juveniles dominated the ichthyoplankton collection in each cruise [49]. Song et al. (2015) reported that the distribution of *Salanx ariakensis* larvae moved up towards shore with the rising tide [50]. We also found that *A. ommaturus*, *A. hexanema*, and *P. fangi* tended to have a higher density at the ebb tide compared with the rising tide (Table 1). Liu et al. (2016) described an increasing abundance of some Gobiidae fishes, which inhabit the junction between brackish and fresh water during ebb tide. They suggested that the freshwater present during ebb tide brought these coastal fishes with weak swimming ability to the outside of the estuary area, with higher abundance in the estuary area, and that the area of the Yangtze River Estuary might be their nursery ground [38].

Regarding the developmental stages of the fish, similar to Ge et al. (2009), we found that the larvae were mainly dominated by individuals in the flexion to juvenile stages in the coastal areas, with very few yolk-sac to preflexion larvae. After reaching a certain body length and developmental stage, the larvae enter the surf zone and coastal areas [51]. Liu et al. (2016) reported that *C. mystus* was distributed in the southeast area of the Yangtze Estuary and that the distribution range was larger and the density was higher during the rising tide. In contrast, they were distributed in the north branch and northeastern area of the Yangtze Estuary at relatively low density during the ebb tide [38]. Together, these findings indicate that the number of variations of the larvae depends on species, developmental stage, and survey location.

In the analysis of the weight of environmental factors, *A. ommaturus*, *P. fangi*, *C. joyneri*, *L. haematocheila*, and *L. japonicus* and the total number of larvae were most influenced by tidal height, with weight percentages (i.e., ability to explain the total deviation contribution rate) of 88.55%, 80.48%, 64.76%, 30.09%, 48.60%, and 50.32%, respectively. The next most important factors were SST and SSS for *C. joyneri* (SST: 25.38%), *L. haematocheila* (SST: 39.52%), and *L. japonicus* (SSS: 41.04%) and total larvae number (SSS: 38.35%). Jiang et al. (2007) found that the mean density of *L. haematocheila* larvae was greater in the stations with water temperatures ranging from 18.8 °C to 19.8 °C, whereas they were less dense at the stations with water temperatures of 21.4 and 17.8 °C [13]. Zeng et al. (2020) reported a significant correlation between mean larval density and water temperature [14], and Chang et al. (2015) found an obvious positive correlation between total number of larvae and water temperature in a mangrove environment [15]. Hou et al. (2018) described obvious seasonal variations in larval number and a positive relationship with water temperature [32]. Hare et al. (2005) suggested that Atlantic menhaden larvae were dominated by residual bottom inflow and wind forcing, summer flounder larvae were dominated by tidal mechanisms, and the importance of tides increased with developmental stages [25]. They concluded that a combination of wind forcing, residual bottom inflow, and selective tidal stream transport were responsible for the migration of larval fishes into Chesapeake Bay [25]. During the survey period, the values of turbidity were > 100 NTU for all larval stages, and the highest value was 180.47 ± 126.15 NTU for groups of 200–500 inds (Table 2). Turbulence might influence the vertical distribution of larvae in unstratified channels [52], and it would especially affect slower-swimming larvae because the turbulent velocities are greater than the swimming speed of the larvae [53].

Except for *L. haematocheila* (26.00%), the importance of turbidity for *A. ommaturus*, *P. fangi*, *C. joyneri*, *L. japonicus*, and total larvae number ranged from 1.78 to 5.95%. The most suitable SST range for *A. ommaturus* and the total larval number was 15.6–16.8 °C, that for *P. fangi*, *L. haematocheila*, and *L. japonicus* was 17.4–20.2 °C, and that for *C. joyneri* was 13.8–16.7 °C. The most suitable SSS ranges for *P. fangi*, *C. joyneri*, *L. japonicus,* and the total larvae number was 27.0–28.8, that for *A. ommaturus* was 28.4–28.8, and that for *L. haematocheila* was 26.6–28.8 psu (Table 3).

**Figure 6:**
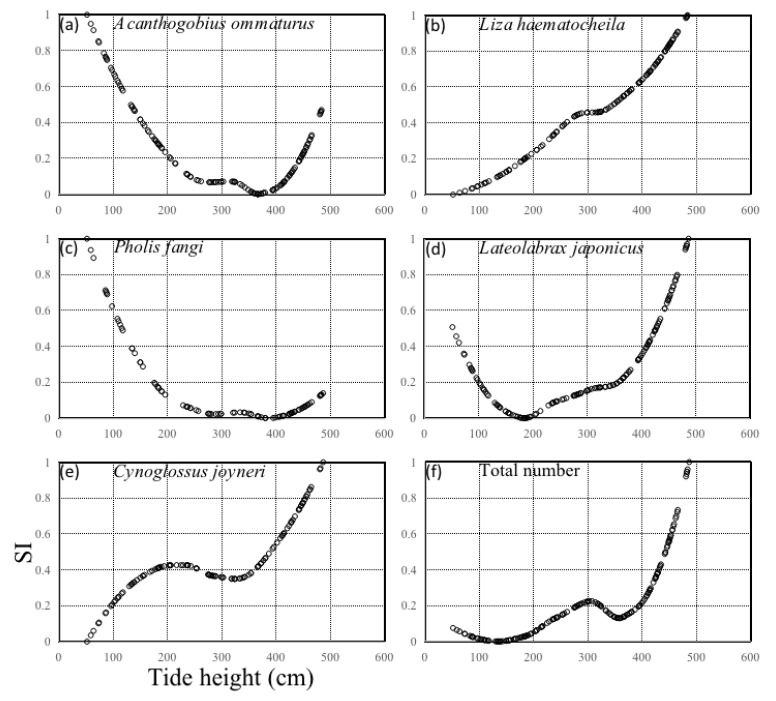
Suitability index (SI) curves denoted by discontinuous hollow circles for (**a**) *Acanthogobius ommaturus*, (**b**) *Liza haematocheila*, (**c**) *Pholis fangi*, (**d**) *Lateolabrax japonicus*, (**e**) *Cynoglossus joyneri*, and (**f**) total larval number corresponding to the tide height (cm) during all of the surveys.

The most suitable turbidity ranges for *A. ommaturus*, *C. joyneri*, *L. japonicus*, and total larvae number were high (482.0–518.0 NTU), that for *P. fangi* was medium (173.0–345.0) NTU, and that for *L. haematocheila* was low (23.9–40.7 NTU) (Table 3, Figure 6). The most suitable tidal height range for *A. ommaturus* and *P. fangi* was 52.0–96.0 cm, and that for *C. joyneri*, *L. haematocheila*, *L. japonicus*, and total larvae number was 421.0–487.0 cm (Table 3). Tidal rhythm is an important environmental factor that affects the drifting pattern of fish juveniles.

In summary, *A. ommaturus* preferred high turbidity, high salinity, and low tidal height; *P. fangi* preferred medium turbidity and low tidal height; *C. joyneri* preferred high turbidity, high salinity, and high tidal height; *L. haematocheila* preferred low turbidity and high tidal height; and *L. japonicus* and total larval number preferred high turbidity and high tidal height.

## 4. Conclusions

We proposed that the Subei Shoal area might provide a migratory channel for fish larvae from the open sea to the coastal area and that these larvae provide part of the fish diet of the red-crowned crane wild population. Fish juveniles use the coastal area as a nursery ground because when the tidal shallows dry up, it is difficult for large predatory fishes to enter the area, which lessens predatory pressure and improves survival rates. Our data about the fish species that support the wild bird population can be used for the sustainable development of the NYBNR area. We suggest that the local government should increase management actions for the stow net fisheries in Jiangsu coastal areas because this fishery poses a threat to the early life stages of economically important fishes and, thus, the diet of the wild bird population.

## Figures and Tables

**Table 1:** Larvae number (LN) and number percentage (NP) of each species and percentage of each developmental stage (yolk-sac: yolk; preflexion: pre; flexion: flex; postflexion: post; juvenile: j; young fish: yg) during the period of day-flood tide, day-ebb tide, night-flood tide, and night-ebb tide of the fish species in the ichthyoplankton samples collected during the survey conducted from 25 April to 10 May 2019.

Latin Name	Type	LN	NP	Yolk	Pre	Flex	Post	J	Yg
*Acanthogobius ommaturus*	Day-Flood	653	24.52%	—	1.43%	5.86%	17.16%	0.08%	—
	Day-Ebb	770	28.91%	—	1.20%	11.19%	16.37%	0.15%	—
	Night-Flood	498	18.70%	—	0.23%	8.26%	9.54%	0.68%	—
	Night-Ebb	742	27.86%	—	0.15%	1.43%	25.16%	1.13%	—
*Acanthopagrus schlegelii*	Day-Flood	—	—	—	—	—	—	—	—
	Day-Ebb	2	100.00%	—	—	—	100.00%	—	—
	Night-Flood	—	—	—	—	—	—	—	—
	Night-Ebb	—	—	—	—	—	—	—	—
*Amblychaeturichthys hexanema*	Day-Flood	22	13.58%	—	—	—	13.58%	—	—
	Day-Ebb	46	28.40%	—	—	—	4.94%	23.46%	—
	Night-Flood	40	24.69%	—	—	—	8.64%	16.05%	—
	Night-Ebb	54	33.33%	—	—	—	16.05%	16.05%	1.23%
*Ammodytes personatus*	Day-Flood	—	—	—	—	—	—	—	—
	Day-Ebb	2	100.00%	—	—	—	100.00%	—	—
	Night-Flood	—	—	—	—	—	—	—	—
	Night-Ebb	—	—	—	—	—	—	—	—
*Anguilla japonica*	Day-Flood	2	100.00%	—	—	—	—	—	100.00%
	Day-Ebb	—	—	—	—	—	—	—	—
	Night-Flood	—	—	—	—	—	—	—	—
	Night-Ebb	—	—	—	—	—	—	—	—
*Callionymus* sp.	Day-Flood	106	33.54%	—	0.63%	3.16%	24.68%	5.06%	—
	Day-Ebb	88	27.85%	—	—	—	12.66%	15.19%	—
	Night-Flood	34	10.76%	—	—	—	9.49%	1.27%	—
	Night-Ebb	88	27.85%	—	1.27%	—	22.15%	4.43%	—
*Clupea pallasii*	Day-Flood	4	100.00%	—	100.00%	—	—	—	—
	Day-Ebb	—	—	—	—	—	—	—	—
	Night-Flood	—	—	—	—	—	—	—	—
	Night-Ebb	—	—	—	—	—	—	—	—
*Cynoglossus joyneri*	Day-Flood	700	13.71%	—	—	0.59%	13.04%	0.08%	—
	Day-Ebb	584	11.44%	—	—	0.55%	10.54%	0.35%	—
	Night-Flood	2562	50.18%	—	—	0.31%	49.82%	0.04%	—
	Night-Ebb	1260	24.68%	—	0.04%	0.59%	23.97%	0.08%	—
*Larimichthys polyactis*	Day-Flood	2336	35.01%	—	0.09%	—	—	34.92%	—
	Day-Ebb	1816	27.22%	—	0.15%	—	—	27.07%	—
	Night-Flood	1274	19.09%	—	—	—	—	19.09%	—
	Night-Ebb	1246	18.68%	—	0.03%	—	—	18.65%	—
*Lateolabrax japonicus*	Day-Flood	2820	43.76%	—	—	—	—	43.76%	—
	Day-Ebb	2118	32.87%	—	—	0.03%	—	32.84%	—
	Night-Flood	654	10.15%	—	—	—	—	10.15%	—
	Night-Ebb	852	13.22%	—	—	—	0.06%	13.16%	—
*Mugil cephalus*	Day-Flood	4	6.25%	—	—	—	—	6.25%	—
	Day-Ebb	14	21.88%	—	—	—	—	21.88%	—
	Night-Flood	30	46.88%	—	—	—	—	46.88%	—
	Night-Ebb	16	25.00%	—	—	—	—	25.00%	—
*Neosalanx anderssoni*	Day-Flood	10	38.46%	—	—	—	—	—	38.46%
	Day-Ebb	12	46.15%	—	—	7.69%	7.69%	—	30.77%
	Night-Flood	—	—	—	—	—	—	—	—
	Night-Ebb	4	15.38%	—	—	—	—	—	15.38%
*Omobranchus elegans*	Day-Flood	—	—	—	—	—	—	—	—
	Day-Ebb	4	100.00%	—	100.00%	—	—	—	—
	Night-Flood	—	—	—	—	—	—	—	—
	Night-Ebb	—	—	—	—	—	—	—	—
*Ophichthyidae* sp.	Day-Flood	2	25.00%	—	25.00%	—	—	—	—
	Day-Ebb	4	50.00%	—	50.00%	—	—	—	—
	Night-Flood	—	—	—	—	—	—	—	—
	Night-Ebb	2	25.00%	—	25.00%	—	—	—	—
*Pholis fangi*	Day-Flood	156	23.56%	—	—	—	1.21%	13.60%	8.76%
	Day-Ebb	268	40.48%	—	—	0.30%	0.60%	11.48%	28.10%
	Night-Flood	64	9.67%	—	—	—	1.21%	3.63%	4.83%
	Night-Ebb	174	26.28%	—	—	—	0.60%	7.25%	18.43%
*Liza haematocheila*	Day-Flood	1074	34.82%	4.09%	26.07%	1.17%	0.39%	3.11%	—
	Day-Ebb	910	29.51%	1.95%	22.11%	1.04%	0.06%	4.35%	—
	Night-Flood	652	21.14%	1.43%	2.59%	0.13%	—	16.99%	—
	Night-Ebb	448	14.53%	0.13%	1.75%	0.39%	—	12.26%	—
*Platycephalus indicus*	Day-Flood	6	42.86%	—	—	—	14.29%	28.57%	—
	Day-Ebb	6	42.86%	—	—	—	14.29%	28.57%	—
	Night-Flood	2	14.29%	—	—	—	14.29%	—	—
	Night-Ebb	—	—	—	—	—	—	—	—
*Scomberomorus niphonius*	Day-Flood	2	100.00%	—	100.00%	—	—	—	—
	Day-Ebb	—	—	—	—	—	—	—	—
	Night-Flood	—	—	—	—	—	—	—	—
	Night-Ebb	—	—	—	—	—	—	—	—
*Stolephorus* sp.	Day-Flood	2	100.00%	—	—	—	100.00%	—	—
	Day-Ebb	—	—	—	—	—	—	—	—
	Night-Flood	—	—	—	—	—	—	—	—
	Night-Ebb	—	—	—	—	—	—	—	—
*Takifugu* sp.	Day-Flood	14	29.17%	—	—	—	29.17%	—	—
	Day-Ebb	8	16.67%	—	—	—	16.67%	—	—
	Night-Flood	12	25.00%	—	—	—	25.00%	—	—
	Night-Ebb	14	29.17%	—	—	—	29.17%	—	—
*Trypauchen vagina*	Day-Flood	—	—	—	—	—	—	—	—
	Day-Ebb	2	50.00%	—	—	—	—	—	50.00%
	Night-Flood	—	—	—	—	—	—	—	—
	Night-Ebb	2	50.00%	—	—	—	—	—	50.00%

**Table 2:** Ratio of the rising tide against ebb tide and the mean value ± standard deviation of the turbidity (NTU) corresponding to the ranges of numbers of individuals (inds).

Individual Number	Rising Tide against Ebb Tide	Turbidity
>500	6:3	114.79 ± 83.73
200–500	16:15	180.47 ± 126.15
100–200	18:18	114.34 ± 93.17
50–100	17:22	105.20 ± 69.53
1–50	23:41	104.11 ± 80.87

**Table 3:** The most suitable value range (SI > 0.7) and the weight percentage (%) of measured environmental variables, including sea surface temperature (SST, °C), sea surface salinity (SSS, psu), turbidity (Tur, NTU), and tidal height (TH, cm), for the fish species *Acanthogobius ommaturus* (AO), *Pholis fangi* (PF), *Cynoglossus joyneri* (CJ), *Liza haematocheila* (LH), and *Lateolabrax japonicus* (LJ) and the total larval number (TN) during the survey period.

	AO	PF	CJ	LH	LJ	TN
SST	0.52%	6.02%	25.38%	39.52%	8.58%	9.36%
	16.0–16.8	18.6–20.1	13.8–16.7	17.6–20.1	17.4–20.2	15.6–16.7
SSS	6.00%	7.55%	6.05%	4.39%	41.04%	38.35%
	28.4–28.8	27.7–28.5	27.3–28.8	26.6–28.8	27.2–28.2	27.0–28.2
Tur	4.93%	5.95%	3.81%	26.00%	1.78%	1.97%
	482.0–518.0	173.0–345.0	482.0–518.0	23.9–40.7	482.0–518.0	482.0–518.0
TH	88.55%	80.48%	64.76%	30.09%	48.60%	50.32%
	52.0–96.0	52.0–87.0	442.0–487.0	421.0–484.0	456.0–487.0	465.0–487.0

## Data Availability

Not applicable.
